# Valuation and Returns of Drug Development Companies: Lessons for Bioentrepreneurs and Investors

**DOI:** 10.1007/s43441-021-00364-y

**Published:** 2022-01-11

**Authors:** Daniel Tobias Michaeli, Hasan Basri Yagmur, Timur Achmadeev, Thomas Michaeli

**Affiliations:** 1 Fifth Department of Medicine, University Hospital Mannheim, Heidelberg University, Mannheim, Germany; 2 TUM School of Management, Technical University of Munich, Munich, Germany; 3 Department of Personalized Oncology, University Hospital Mannheim, Heidelberg University, Mannheim, Germany; 4 Division of Personalized Medical Oncology, German Cancer Research Center (DKFZ), Heidelberg, Germany

**Keywords:** Orphan, Multi-indication, Valuation, Drug development, Investment, Oncology

## Abstract

**Objectives:**

This study evaluates the association of Biopharma company valuation with the lead drug’s development stage, orphan status, number of indications, and disease area. We also estimated annual returns Bioentrepreneurs and investors can expect from founding and investing in drug development ventures.

**Methods:**

SDC Thomson Reuter and S&P Capital IQ were screened for majority acquisitions of US and EU Biopharma companies developing new molecular entities for prescription use (SIC code: 2834). Acquisition data were complemented with drug characteristics extracted from clinicaltrials.gov, the US Food and Drug Administration (FDA), and deal announcements. Thereafter, company valuations were combined with previously published clinical development periods alongside orphan-, indication-, and disease-specific success rates to estimate annual returns for investments in drug developing companies.

**Results:**

Based on a sample of 311 Biopharma acquisitions from 2005 to 2020, companies developing orphan, multi-indication, and oncology drugs were valued significantly higher than their peers during later development stages (*p* < 0.05). We also estimated significantly higher returns for shareholders of companies with orphan relative to non-orphan-designated lead drugs from Phase 1 to FDA approval (46% vs. 12%, *p* < 0.001). Drugs developed across multiple indications also provided higher returns than single-indication agents from Pre-Clinic to FDA approval (21% vs. 11%, *p* < 0.001). Returns for oncology drugs exceeded other disease areas (26% vs. 8%, *p* < 0.001).

**Conclusions:**

Clinical and economic conditions surrounding orphan-designated drugs translate to a favorable financial risk-return profile for Bioentrepreneurs and investors. Bioentrepreneurs must be aware of the upside real option value their multi-indication drug could offer when negotiating acquisition or licensing agreements.

**Supplementary Information:**

The online version contains supplementary material available at 10.1007/s43441-021-00364-y.

## Introduction

Large Biopharma firms are often in the spotlight of public media for bringing novel pharmaceuticals to the market. However, more than half of new drug approvals are developed externally by start-ups or research institutes [1]. While previously published literature focuses on the costs and timelines of the internal research and development (R&D) process [2], in this article, we concentrate on the dynamics of the external drug development process.

A common path for a Biopharma venture to emerge is for scientists to build a business around their novel scientific discovery. These scientists are often subject matter experts in their field. Yet for their venture to succeed they also require managerial competences that extend beyond their scientific work. Specifically, soft skills coupled with managerial principles are crucial to scale a Biopharma venture [3, 4]. Early on Bioentrepreneurs will face the challenge of securing funding for their venture from academic institutions, research grants, and investors. In other words, founders must be able to evaluate both the scientific and financial merits of their discovery. Consequently, in this study, we aim to identify key value drivers of Biopharma ventures based on a cross-sectional sample of 311 Biopharma acquisitions. While it is established that Biopharma firm value is mainly dependent on the lead drug’s development stage [5–9], we also evaluate the association of firm valuation with the lead drug’s FDA orphan designation status, number of indications, molecule type, and disease area.

On the other side, investors and large Biopharma companies must continuously find new ventures to deploy their fund’s capital and commercialize new drugs [10]. They too evaluate both the scientific and financial merits of an investment proposal. Ideally the investment would yield excess—better than average—financial returns. Consequently, the second aim of this paper is to model the risk-return characteristics of the drug development process. We will combine extracted company valuations with success rates and timelines of the drug development process to estimate financial returns. While these are not real returns from a longitudinal dataset of investments, our model permits the identification of industry trends. We will particularly evaluate whether orphan-designated, multi-indication, biologic, and oncology drugs provide excess returns for Bioentrepreneurs and investors.

## Materials and Methods

### Data Collection

SDC Thomson Reuter and S&P Capital IQ were screened for majority acquisitions of Biopharma companies developing new molecular entities (NME) for therapeutic use (SIC code: 2834) from 01.01.2005 to 01.01.2020. Corporations developing generics, reformulations, medical devices, diagnostic substances, over-the-counter medicines, cannabis products, animal therapeutics as well as active pharmaceutical ingredients producers and sales of manufacturing sites were excluded. Only acquisitions with a total deal value beyond $10 million were considered. To exclude mega mergers the sample was limited to targets with a portfolio of less than 10 NME. The geographic location was restricted to targets headquartered in the US or developed European markets. The sample contains both private and public targets.

Financial variables and acquisition characteristics were extracted from SDC Thomson Reuter and S&P Capital IQ. Subsequently, the target’s lead product’s development stage, orphan designation status, number of indications, molecule type, and disease area were obtained from the US FDA database, US SEC filings, clinicaltrials.gov, and deal announcements.

#### Valuation Metrics

Up-front payments, maximum milestone payments (both regulatory and sales), and the overall deal value were obtained from SDC Thomson Reuter and S&P Capital IQ in US dollars at the time of the acquisition. To ensure data validity, all company valuations were cross-checked with US SEC filings and deal announcements, if available. Valuation metrics were adjusted for inflation to 2020 values.

#### Development Stage

The development of human pharmaceutical products can be categorized into five distinct stages: Pre-Clinic, Phase 1, Phase 2, Phase 3, and Approved. We extracted and cross-checked the lead product’s development phase from FDA marketing authorization reports, clinicaltrials.gov, US SEC filings, and deal announcements. Therapeutics in parallel Phase 1/2 (2/3) trials were categorized within the Phase 2 (3) development stage.

#### Orphan Designation

All therapeutics were checked for orphan designations issued by the FDA. Thereby therapeutics were classified as “orphan” and “non-orphan”.

#### Number of Indications

We also extracted the number of indications a therapeutic is being developed for using clinicaltrials.gov. Consequently, therapeutics developed for one disease were categorized as “single-indication” and therapeutics developed for more than one disease were classified as “multi-indication”.

#### Molecule Type

The lead therapeutics molecule type was furthermore classified into “small-molecule” and “biologics or gene/cell therapies”.

#### Disease Area

Lead therapeutics were furthermore classified into disease areas according to the most advanced indication. Categories include oncology, central nervous system (CNS), and others (immunology, infectious disease, cardiovascular, dermatology, internal medicine, ophthalmology).

### Statistical Analysis

First, mean company valuations ± 95% confidence intervals (CI) for orphan designation status, disease area, number of indications, and molecule type were calculated within each development stage. A non-parametric bootstrapped resampling with replacement (1000 iterations) was conducted to calculate mean company valuations and their respective 95%CI in our sample. Thereafter, investment multiples and returns were estimated based on mean company valuations, development stage success rates, and development periods (Table 1).

**Table 1:** Input parameters for the estimation of investment multiples and returns

	Mean	95% CI	Source	σ	α	β	Distribution
Company valuation ($ millions)							
Pre-Clinic	88	(57–119)	^a^	15.65	31.54	2.79	Gamma
Phase 1	399	(211–498)	^a^	66.78	28.17	12.58	Gamma
Phase 2	734	(436–930)	^a^	112.14	31.28	21.84	Gamma
Phase 3	1656	(996–2527)	^a^	369.13	22.77	77.36	Gamma
Approved	2496	(1582–3355)	^a^	432.58	32.57	75.80	Gamma
Success rate (%)							
Pre-Clinic to Phase 1	32.0	(28.8–35.2)	[15]	1.60	271.68	577.32	Beta
Phase 1 to Phase 2	75.8	(68.2–83.4)	[11]	3.79	96.04	30.66	Beta
Phase 2 to Phase 3	55.6	(50.0–61.2)	[11]	2.78	177.04	141.38	Beta
Phase 3 to Approved	67.7	(60.9–74.5)	[11]	3.39	128.52	61.32	Beta
Development period (years)							
Pre-Clinic to Phase 1	1.00	(0.75–1.25)	[16]	0.13	64	0.016	Gamma
Phase 1 to Phase 2	1.50	(1.13–1.88)	[16]	0.19	64	0.023	Gamma
Phase 2 to Phase 3	2.50	(1.88–3.13)	[16]	0.31	64	0.039	Gamma
Phase 3 to Approved	2.50	(1.88–3.13)	[16]	0.31	64	0.039	Gamma

Distinct company valuations and success rates by orphan designation status, number of indications, molecule type, and disease area are enclosed in Supplementary Table e1. Company valuations include up-front and milestone payments and were inflation adjusted to 2020 values

^a^Mean company valuations were calculated from our dataset of 311 Biopharma acquisitions

#### Company Valuation

Mean company valuations were compared by orphan designation status, disease area, number of indications, and molecule type within development stages based on non-parametric bootstrapped *t* tests (resampling of 1000 iterations with replacement). Company valuations were calculated as the sum of the up-front payments and all future milestone payments. Valuations were visualized using beeswarm plots.

Data on company valuations were available for 300 of the 311 collected acquisitions. The analysis of mean valuations and the multiple and return calculation consequently excluded 11 observations to arrive at a final sample of 300 Biopharma acquisitions. No missing data were observed for lead product characteristics, e.g., FDA orphan status, number of indications, molecule type, and disease area.

#### Estimating Investment Multiples

We subsequently estimated multiples Bioentrepreneurs and investors could expect from investments into development stage Biopharma ventures. In the finance industry, investment multiples compare a company’s valuation at the time of sale to a company’s valuation at the time of purchase. Consequently, we estimated investment multiples in the Biopharma context by dividing the mean company valuation of development stage $${\text{Phase }} j$$ by the mean company valuation of development stage $${\text{Phase }} i$$ (Eq. 1). To account for clinical trial failures, investment multiples were adjusted for development stage specific success rates. Clinical success rates were extracted from Wong et al. [11], given that they use the largest sample size, overlap with our study period, and employ the most relevant path-by-path methods – in contrast to other estimations which follow a phase-by-phase methodology [12–14]. Pre-Clinic to clinic success rates were extracted from Takebe et al. who analyzed 798 drug discovery projects in the US [15]. For example, the mean valuation of biopharmaceutical companies with Phase 2 therapeutics ($683 million) was divided by the mean valuation of companies with Phase 1 products ($354 million). This quotient was thereafter adjusted by the success rate to progress from Phase 1 to 2 (75.8%) to arrive at the investment multiple (1.5*x*).

1 $${\text{Multiple}}_{{{\text{Phase }} i {\text{ to }} j}} = \frac{{{\text{Company}} {\text{ Valuation}}_{{{\text{Phase }} j}} }}{{{\text{Company}} {\text{ Valuation}}_{{{\text{Phase }} i}} }}*{\text{Success }} {\text{Rate}}_{{{\text{Phase }} i {\text{ to }} j}}$$

Stage-, indications-, biologic-, disease-, and orphan-specific means for company valuations and success rates were applied to estimate and compare investment multiples and returns within the respective categories. For instance, distinct Phase 1 to 2 (96.1% vs. 75.8%), Phase 2 to 3 (86.1% vs. 55.6%), and Phase 3 to Approved (63.5% vs. 67.7%) success rates were used for orphan vs. non-orphan-designated therapeutics. Similarly, distinct Phase 1 ($227 vs. $370 million), Phase 2 ($744 vs. $673 million), Phase 3 ($2166 vs. $1648 million), and Approved ($3703 vs. $1964 million) company valuations were used for orphan vs. non-orphan therapeutics. Employed success rates and company valuations are enclosed in Supplementary Tables e1 and e3, respectively.

#### Estimating Investment Returns

Finally, annual returns Bioentrepreneurs and investors can expect from investments into development stage Biopharma companies were estimated by linking the previously calculated investment multiple to the mean development time of the respective development stage. Mean development periods were extracted for Pre-Clinic to Phase 1 (1.0 years), Phase 1 to 2 (1.5 years), Phase 2 to 3 (2.5 years), and Phase 3 to Approved (2.5 years) from previous literature [16].

2 $${\text{Investment }} {\text{Return}}_{{{\text{Phase }} i {\text{ to }} j}} = ({\text{Multiple}}_{{{\text{Phase }} i {\text{ to }} j}} )^{{{\text{Mean }} {\text{Development}} {\text{ Time}}_{{{\text{Phase }} i}} }} - 1$$

### Sensitivity Analysis

We conducted a probabilistic sensitivity analysis in Microsoft EXCEL to account for uncertainty surrounding point estimates of company valuation, success rates, and development stage length. Therefore, input parameters for the calculation of investment multiples and returns were drawn by random sampling from their defined distribution displayed in Table 1. Thereby this sampling method permitted the simultaneous variations in considered input parameters. This probabilistic analysis features the simulation of 1,000 investments per variable category.

Data were stored in Microsoft EXCEL and analyzed using STATA SE Version 15.1. For the two-factorial analysis of variance, ANOVA with Dunnett’s/Sidak’s test was applied. A two-tailed probability value < 0.05 was considered significant.

## Results

A total of 2106 unique Biopharma acquisitions were identified in the SDC Thomson Reuter (*n* = 1427) and S&P Capital IQ (*n* = 679) databases between 01.01.2005 and 01.01.2020. Further restricting the search to companies developing NME for human prescription use led to final sample of 311 Biopharma company valuations. Most acquired companies were developing a lead product in Phase 2 (33%) or already commercialized the lead product (20%). Approximately one third of lead products were developed across multiple indications, 21% were classified as biologics or gene/cell therapy, and 21% received FDA orphan designation status. Most acquisitions focused on oncology (30%), CNS (16%), and infectious diseases therapies (11%). More detailed descriptive statistics for the entire sample can be found in Supplementary Table e2.

### Orphan Designation Status

**Fig. 1:**
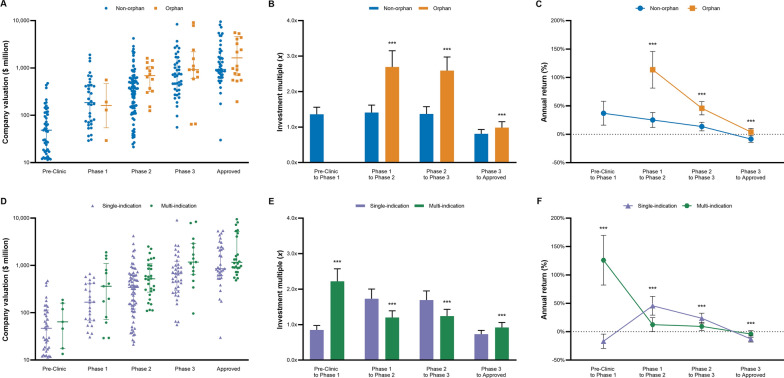
Company valuation, investment multiples, and annual returns by lead drug’s FDA orphan designation status and number of indications. Graphs in the first row compare the valuation (**A**), investment multiples (**B**), and annual returns (**C**) for companies with orphan- and non-orphan-designated lead drugs by development stage. Graphs in the second row compare the valuation (**D**), investment multiples (**E**), and returns (**F**) for companies with multi-indication and single-indication lead drugs by development stage. Valuation data from our sample of 311 Biopharma acquisitions (2005–2020) were inflation adjusted to 2020 values and combined with previously published success rates and development periods to calculate multiples and returns [11, 16]. No valuation data exist for the Pre-Clinic orphan category given that the FDA only issues the orphan designation status after IND approval. *P* values calculated based on ANOVA with Dunnett’s test: *p <* 0.05*, **p *< 0.01*, ***p < 0.001. *FDA* US Food and Drug Administration

Company valuations of orphan and non-orphan-designated therapeutics did not significantly differ for Phase 1 ($227 vs. $370 million, *p* = 0.257), Phase 2 ($744 vs. $673 million, *p* = 0.373), and Phase 3 ($2166 vs. $1648 million, *p* = 0.317; Fig. 1). Company valuation was $3703 million (95%CI 1147–6259) with orphan and $1964 million (95%CI 1331–2597) with non-orphan-designated Approved therapeutics (*p* < 0.05). Estimated multiples of investments in companies with orphan compared to non-orphan-designated therapeutics were significantly higher across all development stages (*p* < 0.001). Similarly, estimated returns of orphan-designated investments significantly exceeded returns of non-orphan investments for Phase 1 to 2 (120% vs. 25%, *p* < 0.001), Phase 2 to 3 (46% vs. 14%, *p* < 0.001) and Phase 3 to Approved (4% vs. − 8%, *p* < 0.001).

Bioentrepreneurs and investors that keep their equity stake in a company with an orphan-designated product from Phase 1 until FDA approval can expect to increase their initial capital by 7.2*x* (95%CI 5.6–9.0) which translates to annual returns of 46% (95%CI 37–56) after adjusting for drug failures (Table 2). In contrast, a similar investment in companies developing non-orphan-designated products would increase the initial capital by only 2.1*x* (95%CI 1.6–2.6, *p* < 0.001) at annual returns of 12% (95%CI 8–16, *p* < 0.001).

**Table 2:** Estimated multiples and returns for investment in drug development Biopharma companies

	Investment multiple (*x*)			Annual return (%)		
	Value	95% CI^a^	*p* value	Value (%)	95% CI^a^	*p* value
FDA orphan designation status						
Orphan	7.2*x*	(5.6–9.0)	< 0.001	46	(37–56)	< 0.001
Non-orphan	2.1*x*	(1.6–2.6)		12	(8–16)	
Number of indications						
Multi-indication	2.9*x*	(2.3–3.7)	< 0.001	21	(15–29)	< 0.001
Single-indication	1.7*x*	(1.3–2.2)		11	(7–14)	
Molecule type						
Biologic OR gene/cell therapy	1.8*x*	(1.4–2.2)	< 0.001	16%	(11–21)	< 0.001
Small-molecule	3.6*x*	(2.8–4.5)		19	(15–25)	
Disease area						
Oncology	4.3*x*	(3.5–5.4)	< 0.001	26	(21–33)	< 0.001
CNS	2.6*x*	(2.0–3.2)	< 0.001	17	(13–22)	< 0.001
Other^b^	1.5*x*	(1.2–1.9)		8	(4–11)	
Overall	2.6*x*	(2.0–3.3)		15	(11–19)	

Multiples and annual returns were estimated assuming an investment horizon from Pre-Clinic until FDA approval. Valuation data from our sample of 311 Biopharma acquisitions (2005–2020) were inflation adjusted to 2020 values and combined with previously published success rates and development periods to calculate multiples and returns [11, 16].

*FDA* US Food and Drug Administration, *CNS* Central nervous system

^a^95% confidence intervals were calculated based on empirical 2.5th and 97.5th percentiles from the conducted sensitivity analysis

^b^The disease category other includes immunology, infectious disease, cardiovascular, dermatology, internal medicine, and ophthalmology

### Number of Indications

Valuations of companies developing multi-indication relative to single-indication lead drugs were significantly higher for Phase 1 ($594 vs. $230 million, *p* < 0.05), Phase 2 ($1058 vs. $522 million, *p* < 0.05), and Approved ($3438 vs. $3438 million, *p* < 0.01), yet not Pre-Clinic ($87 vs. $88 million, *p* = 0.484) and Phase 3 ($2249 vs. $1578 million, *p* = 0.208) development stages. However, estimated multiples of investments in companies with multi-indication relative to single-indication therapeutics were only higher for Pre-Clinic to Phase 1 and Phase 3 to Approved investments periods (*p* < 0.001). For Phase 1 to 2 and Phase 2 to 3, investment multiples of companies with single-indication therapeutics outpace multi-indication products (*p* < 0.001). Similarly, estimated returns were higher for investments in companies with multi-indication relative to single-indication therapeutics for Pre-Clinic to Phase 1 (126% vs. − 16%, *p* < 0.001) and Phase 3 to Approved (− 4% vs. − 12%, *p* < 0.001), yet not for Phase 1 to 2 (12% vs. 45%, *p* < 0.001), Phase 2 to 3 (9% vs. 24%, *p* < 0.001).

Equity stakes in Biopharma companies with multi-indication lead products increased by 2.9*x* (95%CI 2.3–3.7) yielding annualized returns of 21% (95%CI 15–29)—assuming shareholders keep their stakes from Pre-Clinic to FDA approval (Table 2). In contrast, single-indication lead products only provided an overall investment multiple of 1.7*x* (95%CI 1.3–2.2, *p* < 0.001) and annualized returns of 11% (95%CI 7–14, *p* < 0.001).

### Molecule Type

**Fig. 2:**
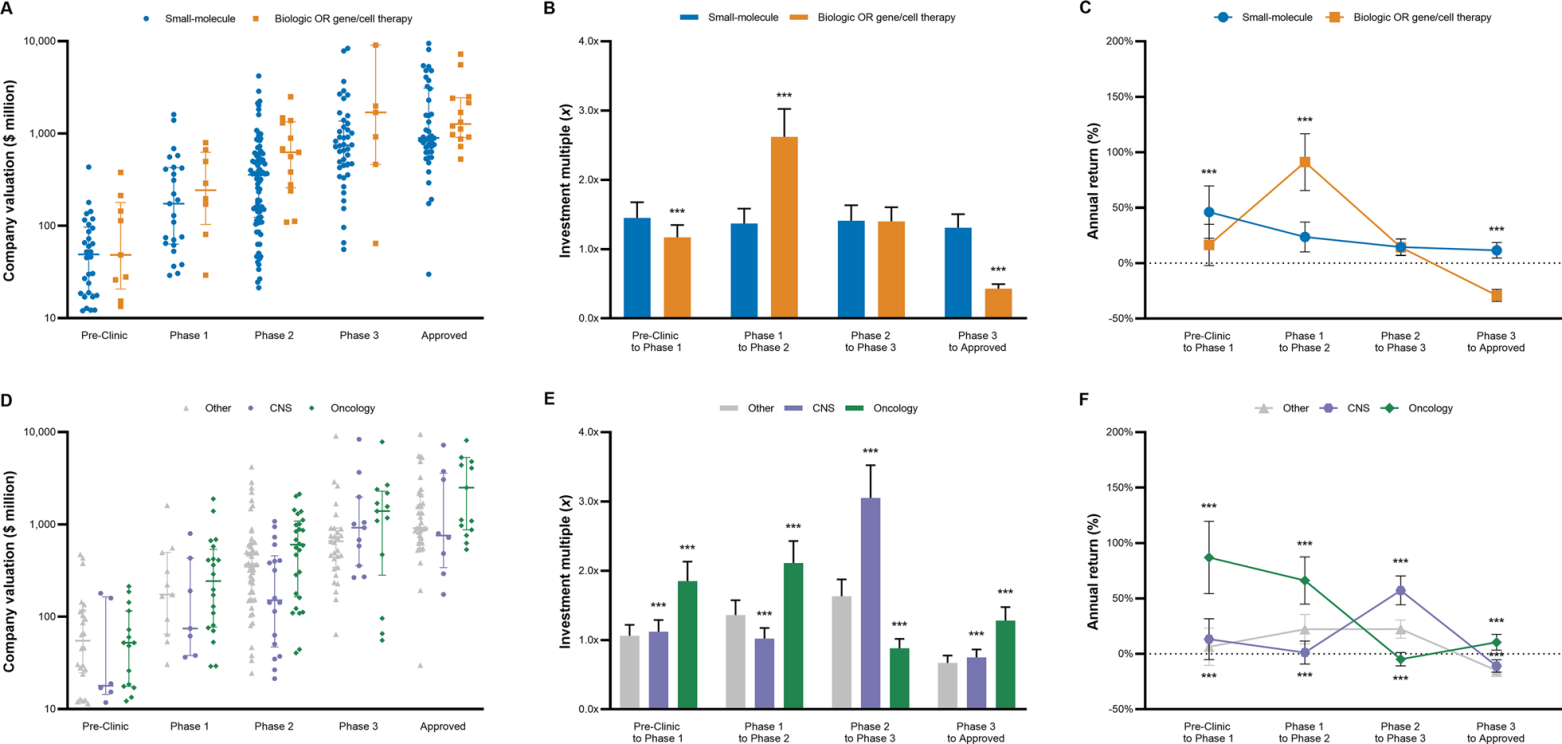
Company valuation, investment multiples, and annual returns by lead drug’s molecule type and disease area. Graphs in the first row compare the valuation (**A**), investment multiples (**B**), and annual returns (**C**) for companies with biologics or gene/cell therapies and small-molecule lead drugs by development stage. Graphs in the second row compare the valuation (**D**), investment multiples (**E**), and returns (**F**) for companies with lead drugs in oncology, CNS, and other disease areas by development stage. Valuation data from our sample of 311 Biopharma acquisitions (2005–2020) were inflation adjusted to 2020 values and combined with previously published success rates and development periods to calculate multiples and returns [11, 16]. The disease category other includes immunology, infectious disease, cardiovascular, dermatology, internal medicine, and ophthalmology. *P* values calculated based on ANOVA with Dunnett’s test (Sidak’s test for disease area): *p < 0.05, **p < 0.01, ***p < 0.001. *CNS* central nervous system

Companies developing biologics or gene and cell therapies were valued higher relative to small-molecules during Pre-Clinic ($109 vs. $71 million, *p* < 0.05), Phase 1 ($341 vs. $325 million, *p* = 0.139), Phase 2 ($811 vs. $517 million, *p* < 0.05), and Phase 3 ($2,249 vs. $1,578 million, *p* = 0.103) yet not Approved development stages ($2088 vs. $2105 million, *p* = 0.161). However, estimated returns for investments in companies with biologics or gene and cell therapies were lower for Pre-Clinic to Phase 1 (18% vs. 46%, *p* < 0.001), higher for Phase 1 to 2 (93% vs. 23%, *p* < 0.001), the same for Phase 2 to 3 (14% vs. 15%, *p* = 0.53), and lower for Phase 3 to Approved (− 29% vs. 11%, *p* < 0.001) development stages relative to small-molecules (Fig. 2).

Bioentrepreneurs and investors that keep their equity stake in a company with a biologic or gene therapeutic lead product from Phase 1 until FDA approval can expect to increase their initial capital by 1.8*x* (95%CI 1.4–2.2) which translates to annual returns of 16% (95%CI 11–21) after adjusting for drug failures (Table 2). In contrast, a similar investment in companies developing small-molecule drugs would see their capital grow by 3.6*x* (95%CI 2.8–4.5, *p* < 0.001) at annual returns of 19% (95%CI 15–25, *p* < 0.001).

### Disease Area

Companies with oncology drugs were valued significantly higher during Phase 2 development ($1068, *p* < 0.05), while companies with CNS lead drugs ($314 million, *p* < 0.001) were valued lower than other companies ($607 million). Simulated returns of investments into oncology relative to CNS lead drugs were significantly higher for Pre-Clinic to Phase 1 (89% vs. 13%, *p* < 0.001), Phase 1 to Phase 2 (66% vs. 2%, *p* < 0.001), Phase 3 to Approved (10% vs. − 11%, *p* < 0.001), yet not Phase 2 to 3 (− 5% vs. 57%, *p* < 0.001).

Assuming an investment horizon from Pre-Clinic to FDA approval, founders and capital providers of Biopharma companies with oncology therapeutics can expect to increase their invested capital to a greater extent compared to companies developing CNS and other therapeutic agents (*p* < 0.001). Estimated annual returns from Pre-Clinic to FDA approval were 26% (95%CI 21–33) for oncology, 17% (95%CI 13–22) for CNS, and 8% (95%CI 4–11) for other lead therapeutics (Table 2).

## Discussion and Conclusion

This study first assessed the association of Biopharma company valuation with development stage, FDA orphan designation status, number of indications, molecule type, and disease area based on a sample of 311 Biopharma acquisition from 2005 to 2020. Thereafter, company valuations were combined with previously published clinical development periods and success rates to estimate investment multiples and annual returns. Orphan-designated (46%), oncology (26%), CNS (17%), multi-indication (21%), and small-molecule (19%) drugs were projected to provide significantly higher than average (15%) annual returns to company shareholders holding equity stakes from Pre-Clinic to FDA approval (*p* < 0.001). These results provide Bioentrepreneurs with first insights into the valuation and potential of their ventures. On the other side, results educate financial and strategic financiers on investment opportunities with favorable risk-return profiles.

Our dataset illustrates that Biopharma company valuation is mainly driven by the lead product’s development stage. This is in line with previous studies investigating Biopharma acquisitions and therapeutic licensing agreements [6, 7, 17]. Our calculations also demonstrate downward sloping annual returns for advanced drug development stages (Supplementary Figure e1). Assuming annual returns are correlated to an investment’s risk, this result implies risk is unequally distributed across the drug development process. This observation is coherent with expectations given that the pre-clinical drug discovery process and early clinical development are associated with several unknown factors and incur higher attrition rates than late-stage clinical development [11, 16]. The more pre-clinical and clinical data are available on a drug, the lower the uncertainty and also the risk of failure, resulting in diminished returns. Overall, we calculated annual returns of 15% throughout the drug development process. This is slightly higher than previous estimates of mean industry returns for new drug introductions of 11.5% [18]. These estimates are based on new drug launches from the 1990s in large, publically listed Biopharma companies, whereas our estimate is derived from riskier acquisitions of private and public small to medium-sized Biopharma ventures from 2005 to 2020.

In contrast to Rooswinkel et al. [19], our dataset of 311 Biopharma acquisitions demonstrates that late-stage valuations were significantly higher for companies developing orphan relative to non-orphan lead products. Accordingly, we also estimated higher investment multiples and returns for shareholders of companies with orphan relative non-orphan-designated lead products from Phase 1 to FDA approval (46% vs. 12%, *p* < 0.001). Several reasons may help to explain this observation. Orphan drugs possess favorable economics given the lower clinical and FDA attrition rates, firmer and prolonged market exclusivity periods, financial R&D incentives, expedited development and FDA approval periods, swift market penetration, and greater reimbursement prices [12, 20, 21]. Consequently, Bioentrepreneurs developing orphan-designated products confidently negotiate higher company valuations with financial investors. On the other side, investors may particularly seek to invest in orphan therapeutic as their unique economics translate to a favorable financial risk-return profile.

In 2019, the 10 highest grossing drugs were all authorized and commercialized across more than one therapeutic indication. Accordingly, results demonstrate that late-stage valuations were significantly higher for companies developing multi-indication relative to single-indication lead products. Similarly, we calculated that investment in pre-clinical companies with multi-indication lead products yield annual returns of 21% until FDA approval, whereas single-indication products only provide a return of 11% per year. Multi-indication therapeutics provide companies with an upside option to develop and commercialize the drug across several diseases if it proofs to be safe and efficacious in initial trials. Additionally, early drug discovery efforts and timelines can be dynamically offset as they are only undertaken once per drug [22]. Furthermore, companies may engage in the sequencing of indications according to clinical benefit and patient population in order to establish higher drug prices and revenues under prevailing single price policies [23–25]. Consequently, Bioentrepreneurs must be aware of the upside real option value their therapeutic could offer to large Biopharma corporations when engaging in acquisition or licensing deals. Oppositely, strategic investors can benefit from the large market potential and favorable risk-return profile of multi-indication products.

The collected dataset of 311 Biopharma acquisitions did not reveal any significant valuation difference for companies with small-molecules compared to biologic or gene/cell therapy lead products. Nonetheless, we estimated that shareholders of Biopharma companies developing small-molecules can expect higher returns than companies developing biologics or gene/cell therapies (19% vs. 16%, *p* < 0.001). This result is surprising as one might expect that the often superior clinical safety and efficacy profile of biologics and gene/cell therapeutics—which frequently permit the treatment or even cure of previously untreatable disease—would translate into a greater economic and financial benefit [12, 26–28]. Nonetheless, these clinical benefits could be offset by large initial investment outlay, higher production cost, inconvenient administration routes, and reimbursement hurdles often faced by biologics and gene/cell therapies [29, 30]. Consequently, purely financial investors should be cautious about venture opportunities of biologics and gene/cell therapies. In contrary, large Biopharma corporations could potentially benefit from strategic investments into such technologies as this provides them access to the capabilities and human capital of the innovative venture [31].

Small and large Biopharma ventures alike frequently focus their R&D efforts on disease areas with a high unmet patient need—oncology and CNS [7, 32]. However, our dataset did not exhibit a significant difference in company valuation according to therapeutic area. Nonetheless, our calculations show higher returns for shareholders with oncology (26%) and CNS (17%) products relative to other (8%) therapeutic areas (*p* < 0.001) from Pre-Clinic to FDA approval. This discrepancy might have multifactorial reasons. From a clinical perspective, oncology drugs offer a survival benefit for patients and often also improve quality of life [33]. In contrast, drugs in other areas mostly improve patients’ quality of life without a proven effect on overall survival. Consequently, higher annual treatment costs and drug prices are observed for cancer drugs [34]. While some authors argue that these high prices are justified by providing clinical value to patients in areas of high unmet, Prasad et al. argue that the observed prices cannot be explained by rational arguments such as R&D costs and the absence of treatment alternatives [35]. As a result, Biopharma companies stand to make a profit on this mismatch between high prices yet arguably marginal benefit for patients [36]. Additionally, many cancer drugs are launched for rare diseases, whereby Biopharma companies realize the previously discussed favorable economics of the FDA orphan designation status. Moreover, Biopharma companies could profit from launching me-too drugs which are priced similar to first-in-class agents yet consume less R&D resources [37]. A combination of all these factors help to explain the estimated higher returns of investments in companies developing oncology drugs.

## Strengths & Limitations

Strengths of this study include its large sample size, uniquely detailed drug characteristics, and employed modeling technique. However, this study also has several limitations. First, non-disclosed information may bias results. Specifically, undisclosed acquisitions of very early-stage pre-clinical corporations may not be released and thereby overestimate company valuations of the pre-clinical development stage, which in turn cause returns to be underestimated.

Second, investment multiples and returns were estimated based on a cross-section of company valuations. Detailed longitudinal data entailing a company’s valuation alongside its drug’s development stage and characteristics would be necessary to correctly evaluate real returns received by Bioentrepreneurs and investors. However, given that this information is not publicly disclosed our employed methodology of combining company valuation with success rates and development periods may offer first insights into the investment characteristics of this industry.

Third, our analysis only captures value arising from the lead product. Biopharma companies in our sample frequently develop numerous drugs at the same time. Therefore, a more detailed modeling technique capturing the further product portfolio’s value is necessary to more realistically estimate investment multiples and returns. However, our assumption of only considering the development stage of the lead product may account for most of a company’s value given that products usually share a similar technology or mechanism of action. Consequently, if the lead product fails a development stage, the likelihood of the other products failing could be similarly high. Moreover, early-stage developments of additional indications for a company’s lead drug may not be disclosed. Therefore, data on the total number of indications may have been missing and consequently bias results. Nevertheless, this effect is assumed to be minor given that a company’s main value driver remains the lead drug’s first (most advanced) indication.

Finally, our methodology projected overall negative returns for the Phase 3 to FDA approval investment period. Valuation of companies in the Approved development stage also includes products that have already been on the market for some years and could consequently face loss of exclusivity. As a result, our sample may underestimate Biopharma company valuation right after FDA approval, which thereby also underestimates returns.

## Supplementary Information

Below is the link to the electronic supplementary material.

Supplementary file1 (PDF 519 kb)
