# Supplementary material for: Valuation and Returns of Drug Development Companies: Lessons for Bioentrepreneurs and Investors
Source: Ther Innov Regul Sci. 2022 Jan 11;56(2):313–22. doi: 10.1007/s43441-021-00364-y (PMC8854317; doi:10.1007/s43441-021-00364-y)
Supplement: Supplementary file 1 — Supplementary file1 (PDF 519 kb) [file 43441_2021_364_MOESM1_ESM.pdf]

Supplementary Online Content

Michaeli DT, Yagmur HB, Achmadeev T, Michaeli T. Valuation and Returns of Drug Development Companies: Lessons for Bioentrepreneurs and Investors. Therapeutic Innovation & Regulatory Science. doi:10.1007/s43441-021-00364-y

Table e1 Drug development success rates by subgroups

Table e2 Descriptive statistics for the entire sample of Biopharma acquisitions

Table e3 Stage-specific Biopharma company valuation (in million US dollars) by FDA orphan status, number of indications, molecule type, and disease area

Figure e1 Company valuation, investment multiples, and annual returns, by development stage for the entire sample

This supplementary material has been provided by the authors to give readers additional information about their work.

	Overall	Orphan	Oncology	CNS	Small-molecule	Biologic OR gene/cell therapy	Single-indication	Multi-indication
Phase 1 to 2	75.8%	96.1%	78.7%	75.0%	74.5%	84.4%	75.8%	66.4%
Phase 2 to 3	55.6%	86.1%	53.9%	54.5%	52.5%	60.1%	55.6%	58.3%
Phase 3 to FDA Approval	67.7%	63.5%	48.5%	63.0%	71.0%	80.4%	67.7%	59.0%
**Phase 1 to FDA Approval**	**28.5%**	**52.5%**	**20.6%**	**25.8%**	**27.8%**	**40.8%**	**28.5%**	**22.8%**

Table e1 Drug development success rates by subgroups

Data extracted from Wong et al. [1]. For the purposes of our analyses, success rates for orphan, small-molecule, and biologic drugs were adjusted to lead drugs only [1, 2]. *CNS* central nervous system, *FDA* US Food and Drug Administration.

	N	Mean	Median	SD	Min.	Max.	Sum
**A) Valuation**							
Up-front payment	303	958	257	2,270	0	22,434	290,417
Milestone payment	290	159	0	330	0	2,945	46,144
Total deal value	300	1,119	458	2,285	12	22,434	335,662
**B) Lead drug**							
Development stage							
Pre-Clinic	311	0.15	0.00	0.36	0	1	46
Phase 1	311	0.13	0.00	0.34	0	1	40
Phase 2	311	0.33	0.00	0.47	0	1	103
Phase 3	311	0.19	0.00	0.39	0	1	59
Approved	311	0.20	0.00	0.40	0	1	63
No. of indications	311	1.83	1.00	1.88	1	16	569
Biologic OR gene/cell therapy	311	0.21	0.00	0.41	0	1	66
Disease area							
Oncology	311	0.30	0.00	0.46	0	1	93
CNS	311	0.16	0.00	0.37	0	1	51
Others	311	0.53	0.00	0.50	0	1	167
FDA orphan designation	311	0.16	0.00	0.37	0	1	51
**C) Other products**							
Total no. of drugs	311	2.96	2.00	2.19	1	10	921
Average no. of indications	311	1.46	1.00	1.09	1	12	455
**D) Acquisition characteristics**							
Target headquarter US	311	0.76	1.00	0.43	0	1	235
Spin-off / single drug deal	311	0.11	0.00	0.31	0	1	34

Table e2 Descriptive statistics for the entire sample of Biopharma acquisitions

All valuation metrics are inflation adjusted to 2020. *CNS* central nervous system, *FDA* US Food and Drug Administration, *SD* standard deviation.

	Pre-Clinic		Phase 1		Phase 2		Phase 3		Approved	
	Mean	*P* Value	Mean	*P* Value	Mean	*P* Value	Mean	*P* Value	Mean	*P* Value
**FDA orphan designation status**										
Orphan	NA[a]	*NA* [a]	227	*p=0.257*	744	*p=0.373*	2,166	*p=0.317*	3,703	*p<0.05*
Non-orphan	88		370		673		1,648		1,964	
**No. of indications**										
Multi-indication	87	*p=0.484*	594	*p<0.05*	1,058	*p<0.05*	2,249	*p=0.208*	3,438	*p<0.01*
Single-indication	88		230		522		1,578		1,670	
**Molecule type**										
Small-molecule	71	*p<0.05*	325	*p=0.139*	517	*p<0.05*	1,536	*p=0.103*	2,105	*p=0.161*
Biologic OR gene/cell therapy	109		341		811		3,759		2,088	
**Disease area**										
Oncology	70	*p=0.176*	403	*p=0.242*	1,068	*p<0.05*	1,747	*p=0.490*	4,561	*p<0.01*
CNS	67	*p=0.346*	233	*p=0.204*	314	*p<0.001*	1,740	*p=0.490*	2,066	*p=0.372*
Other [b]	104		343		607		1,775		1,747	
**Mean**	**99**		**479**		**856**		**2,255**		**1,964**	

Table e3 Stage-specific Biopharma company valuation (in million US dollars) by FDA orphan status, number of indications, molecule type, and disease area

All valuation metrics are inflation adjusted to 2020. *P* Values calculated based on nonparametric bootstrapped *t* tests (resampling of 1,000 iterations with replacement). *CNS* central nervous system, *FDA* US Food and Drug Administration, *NA* not applicable.

[a] No valuation data exists for the Pre-Clinic orphan category given that the FDA only issues the orphan designation status after IND approval.
[b] The disease category "other" includes immunology, infectious disease, cardiovascular, dermatology, internal medicine, ophthalmology.

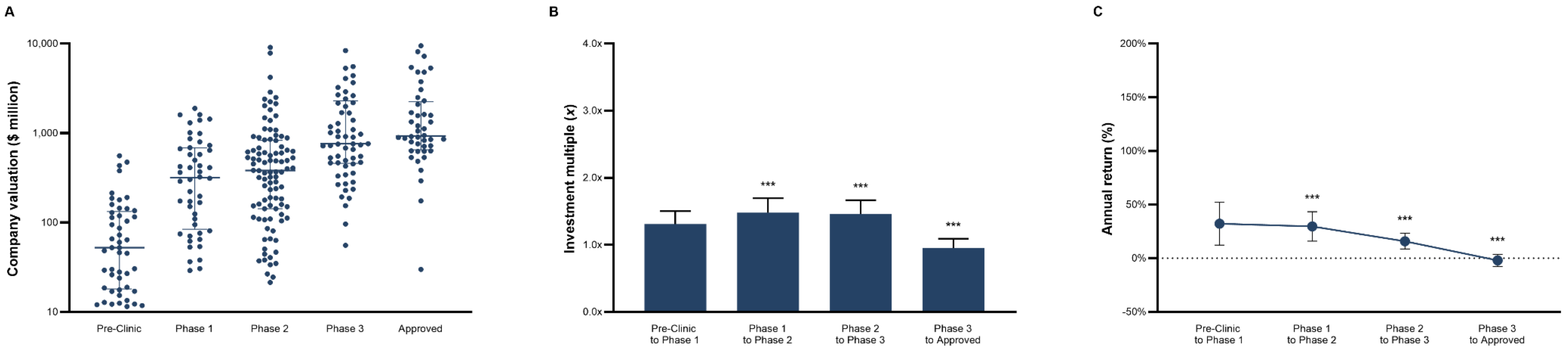

Figure e1 Company valuation (**A**), investment multiples (**B**), and annual returns (**C**) by development stage for the entire sample

All valuation metrics are inflation adjusted to 2020. *P* Values calculated based on ANOVA with Dunnett's test compared to Pre-Clinic to Phase 1: *$p < 0.05$, **$p < 0.01$, ***$p < 0.001$.
